# (*E*)-Methyl *N*′-(2-hydr­oxy-3-methoxy­benzyl­idene)hydrazinecarboxyl­ate

**DOI:** 10.1107/S1600536809021631

**Published:** 2009-06-10

**Authors:** Lu-Ping Lv, Wen-Bo Yu, Wei-Wei Li, Yong-Zhao Zhang, Xian-Chao Hu

**Affiliations:** a Department of Chemical Engineering, Hangzhou Vocational and Technical College, Hangzhou 310018, People’s Republic of China; b Research Center of Analysis and Measurement, Zhejiang University of Technology, Hangzhou 310014, People’s Republic of China

## Abstract

The title compound, C_10_H_12_N_2_O_4_, adopts a *trans* configuration with respect to the C=N double bond. The non-H atoms of the mol­ecule are essentially coplanar, with a maximum deviation of 0.015 (2) Å. An intra­molecular O—H⋯N inter­action is observed. In the crystal structure, the mol­ecules are linked into a two-dimensional network parallel to the *ac* plane by N—H⋯O hydrogen bonds involving the meth­oxy O atom and by two C—H⋯O hydrogen bonds involving the carbonyl O atom. In addition, an intermolecular C—H⋯π inter­action is observed.

## Related literature

For general background to the properties of benzaldehyde­hydrazone derivatives, see: Parashar *et al.* (1988 ▶); Hadjoudis *et al.* (1987 ▶); Borg *et al.* (1999 ▶). For the use of metal complexes of Schiff bases as model compounds of active centres in various proteins and enzymes, see: Kahwa *et al.* (1986 ▶); Santos *et al.* (2001 ▶). For a related structure, see: Shang *et al.* (2007 ▶).
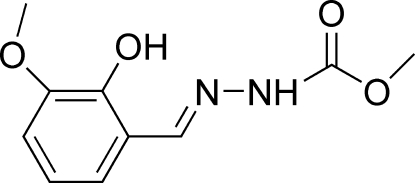


## Experimental

### 

#### Crystal data

C_10_H_12_N_2_O_4_
*M* *_r_* = 224.22
Monoclinic, 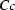

*a* = 11.4348 (13) Å
*b* = 14.8717 (18) Å
*c* = 6.3508 (8) Å
β = 98.538 (4)°
*V* = 1068.0 (2) Å^3^
*Z* = 4
Mo *K*α radiation
μ = 0.11 mm^−1^
*T* = 223 K
0.24 × 0.22 × 0.17 mm

#### Data collection

Bruker SMART CCD area-detector diffractometer
Absorption correction: multi-scan (*SADABS*; Bruker, 2002 ▶) *T*_min_ = 0.975, *T*_max_ = 0.985
5851 measured reflections
1049 independent reflections
948 reflections with *I* > 2σ(*I*)
*R*_int_ = 0.021

#### Refinement

*R*[*F* ^2^ > 2σ(*F* ^2^)] = 0.029
*wR*(*F* ^2^) = 0.075
*S* = 1.11
1049 reflections
148 parameters
2 restraints
H-atom parameters constrained
Δρ_max_ = 0.11 e Å^−3^
Δρ_min_ = −0.13 e Å^−3^

### 

Data collection: *SMART* (Bruker, 2002 ▶); cell refinement: *SAINT* (Bruker, 2002 ▶); data reduction: *SAINT*; program(s) used to solve structure: *SHELXS97* (Sheldrick, 2008 ▶); program(s) used to refine structure: *SHELXL97* (Sheldrick, 2008 ▶); molecular graphics: *SHELXTL* (Sheldrick, 2008 ▶); software used to prepare material for publication: *SHELXTL*.

## Supplementary Material

Crystal structure: contains datablocks I, global. DOI: 10.1107/S1600536809021631/ci2816sup1.cif

Structure factors: contains datablocks I. DOI: 10.1107/S1600536809021631/ci2816Isup2.hkl

Additional supplementary materials:  crystallographic information; 3D view; checkCIF report

## Figures and Tables

**Table 1:** Hydrogen-bond geometry (Å, °)

*D*—H⋯*A*	*D*—H	H⋯*A*	*D*⋯*A*	*D*—H⋯*A*
O1—H1⋯N1	0.82	1.93	2.645 (2)	145
N2—H2⋯O2^i^	0.86	2.42	3.022 (2)	127
C5—H5⋯O3^ii^	0.93	2.49	3.320 (2)	149
C7—H7⋯O3^ii^	0.93	2.45	3.291 (2)	150
C3—H3⋯*Cg*1^iii^	0.93	2.85	3.606 (2)	139

Symmetry codes: (i) 
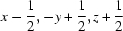
; (ii) 
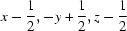
; (iii) 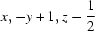. *Cg*1 is the centroid of the C1–C6 ring.

**Fig. 1.**
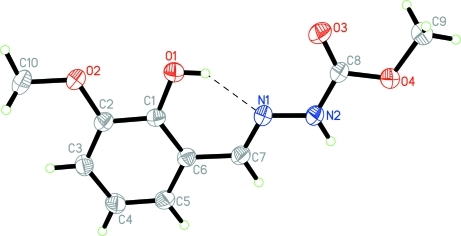
The molecular structure of the title compound. Displacement ellipsoids are drawn at the 30% probability level. The dashed line indicates a hydrogen bond.

**Fig. 2.**
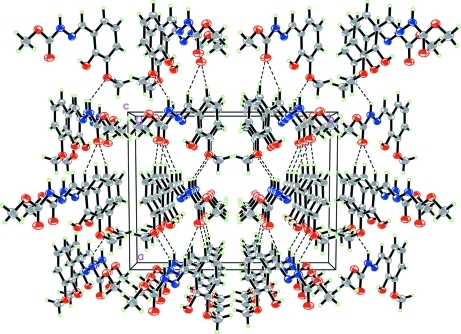
Crystal packing of the title compound. Hydrogen bonds are shown as dashed lines.

## supplementary crystallographic information

### Comment

Benzaldehydehydrazone derivatives have attracted much attention due to their pharmacological activity (Parashar *et al.*, 1988) and their photochromic properties (Hadjoudis *et al.*, 1987). They are important intermediates of 1,3,4-oxadiazoles, which have been reported to be versatile compounds with many interesting properties (Borg *et al.*, 1999). Metal complexes based on Schiff bases have received considerable attention because they can be utilized as model compounds of active centres in various proteins and enzymes (Kahwa *et al.*, 1986; Santos *et al.*, 2001). We report here the crystal structure of the title compound.

The title molecule adopts a *trans* configuration with respect to the C═N bond. The non-hydrogen atoms of the molecule are essentially coplanar, with a maximum deviation of 0.015 (2) Å for atom C(7). The bond lengths and angles are comparable to those observed for methyl*N*'-[(*E*)-4-methoxybenzylidene]hydrazinecarboxylate (Shang *et al.*, 2007). An intramolecular O—H···N interaction is observed.

In the crystal structure, the molecules are linked into a two-dimensional network parallel to the *ac* plane by N—H···O and C—H···O hydrogen bonds (Table 1 and Fig.2). In addition, C—H···π interactions are observed.

### Experimental

2-Hydroxy-3-methoxybenzaldehyde (1.52 g, 0.01 mol) and methyl hydrazinecarboxylate (0.90g, 0.01 mol) were dissolved in stirred methanol (20 ml) and left for 3.5 h at room temperature. The resulting solid was filtered off and recrystallized from ethanol to give the title compound in 90% yield. Single crystals suitable for X-ray analysis were obtained by slow evaporation of an ethanol solution at room temperature (m.p. 415–418 K).

### Refinement

H atoms were positioned geometrically (O-H = 0.82 Å, N-H = 0.86 Å and C-H = 0.93 or 0.96 Å) and refined using a riding model, with *U*_iso_(H) = 1.2*U*_eq_(C,N) and 1.5*U*_eq_(C_methyl_). In the absence of significant anomalous scattering effects, Friedel pairs were averaged.

### Crystal data

**Table:**

C_10_H_12_N_2_O_4_	*F*(000) = 472
*M**_r_* = 224.22	*D*_x_ = 1.394 Mg m^−^^3^
Monoclinic, *C**c*	Mo *K*α radiation, λ = 0.71073 Å
Hall symbol: C -2yc	Cell parameters from 1049 reflections
*a* = 11.4348 (13) Å	θ = 2.3–26.0°
*b* = 14.8717 (18) Å	µ = 0.11 mm^−^^1^
*c* = 6.3508 (8) Å	*T* = 223 K
β = 98.538 (4)°	Block, colourles
*V* = 1068.0 (2) Å^3^	0.24 × 0.22 × 0.17 mm
*Z* = 4	

### Data collection

**Table:**

Bruker SMART CCD area-detector diffractometer	1049 independent reflections
Radiation source: fine-focus sealed tube	948 reflections with *I* > 2σ(*I*)
graphite	*R*_int_ = 0.021
φ and ω scans	θ_max_ = 26.0°, θ_min_ = 2.3°
Absorption correction: multi-scan (*SADABS*; Bruker, 2002)	*h* = −14→14
*T*_min_ = 0.975, *T*_max_ = 0.985	*k* = −16→18
5851 measured reflections	*l* = −7→7

### Refinement

**Table:**

Refinement on *F*^2^	Primary atom site location: structure-invariant direct methods
Least-squares matrix: full	Secondary atom site location: difference Fourier map
*R*[*F*^2^ > 2σ(*F*^2^)] = 0.029	Hydrogen site location: inferred from neighbouring sites
*wR*(*F*^2^) = 0.075	H-atom parameters constrained
*S* = 1.11	*w* = 1/[σ^2^(*F*_o_^2^) + (0.0409*P*)^2^ + 0.1415*P*] where *P* = (*F*_o_^2^ + 2*F*_c_^2^)/3
1049 reflections	(Δ/σ)_max_ = 0.003
148 parameters	Δρ_max_ = 0.11 e Å^−^^3^
2 restraints	Δρ_min_ = −0.13 e Å^−^^3^

### Special details

**Table:**

Geometry. All esds (except the esd in the dihedral angle between two l.s. planes) are estimated using the full covariance matrix. The cell esds are taken into account individually in the estimation of esds in distances, angles and torsion angles; correlations between esds in cell parameters are only used when they are defined by crystal symmetry. An approximate (isotropic) treatment of cell esds is used for estimating esds involving l.s. planes.
Refinement. Refinement of F^2^ against ALL reflections. The weighted R-factor wR and goodness of fit S are based on F^2^, conventional R-factors R are based on F, with F set to zero for negative F^2^. The threshold expression of F^2^ > 2sigma(F^2^) is used only for calculating R-factors(gt) etc. and is not relevant to the choice of reflections for refinement. R-factors based on F^2^ are statistically about twice as large as those based on F, and R- factors based on ALL data will be even larger.

### Fractional atomic coordinates and isotropic or equivalent isotropic displacement parameters (Å^2^)

**Table:**

	*x*	*y*	*z*	*U*_iso_*/*U*_eq_	
C1	0.11401 (17)	0.33419 (13)	0.6468 (3)	0.0412 (5)	
C2	0.14440 (19)	0.38079 (15)	0.4705 (4)	0.0446 (5)	
C3	0.0578 (2)	0.42357 (16)	0.3309 (4)	0.0513 (6)	
H3	0.0779	0.4550	0.2149	0.062*	
C4	−0.0596 (2)	0.4195 (2)	0.3646 (4)	0.0584 (6)	
H4	−0.1176	0.4484	0.2704	0.070*	
C5	−0.0906 (2)	0.37377 (16)	0.5339 (4)	0.0528 (6)	
H5	−0.1696	0.3711	0.5531	0.063*	
C6	−0.00420 (19)	0.33056 (15)	0.6795 (3)	0.0436 (5)	
C7	−0.04116 (18)	0.28326 (16)	0.8601 (3)	0.0469 (5)	
H7	−0.1203	0.2836	0.8788	0.056*	
C8	0.07691 (19)	0.15569 (15)	1.3010 (3)	0.0451 (5)	
C9	0.1022 (3)	0.0689 (2)	1.6128 (5)	0.0690 (8)	
H9A	0.1535	0.0304	1.5467	0.103*	
H9B	0.0569	0.0334	1.6977	0.103*	
H9C	0.1487	0.1118	1.7019	0.103*	
C10	0.2979 (3)	0.4277 (2)	0.2805 (5)	0.0767 (9)	
H10A	0.2597	0.4021	0.1495	0.115*	
H10B	0.3821	0.4231	0.2868	0.115*	
H10C	0.2760	0.4898	0.2874	0.115*	
N1	0.03469 (15)	0.24138 (13)	0.9926 (3)	0.0462 (4)	
N2	−0.00459 (16)	0.19813 (14)	1.1600 (3)	0.0502 (5)	
H2	−0.0782	0.1981	1.1743	0.060*	
O1	0.20402 (14)	0.29467 (12)	0.7790 (3)	0.0553 (4)	
H1	0.1776	0.2697	0.8770	0.083*	
O2	0.26239 (14)	0.38043 (11)	0.4552 (3)	0.0561 (4)	
O3	0.18042 (15)	0.15245 (13)	1.2939 (3)	0.0660 (5)	
O4	0.02356 (14)	0.11544 (12)	1.4512 (3)	0.0572 (5)	

### Atomic displacement parameters (Å^2^)

**Table:**

	*U*^11^	*U*^22^	*U*^33^	*U*^12^	*U*^13^	*U*^23^
C1	0.0391 (10)	0.0391 (12)	0.0450 (11)	0.0002 (8)	0.0049 (9)	−0.0029 (10)
C2	0.0418 (10)	0.0435 (12)	0.0494 (12)	−0.0029 (8)	0.0099 (9)	−0.0048 (9)
C3	0.0525 (13)	0.0533 (14)	0.0481 (14)	−0.0039 (10)	0.0069 (10)	0.0079 (11)
C4	0.0442 (11)	0.0696 (17)	0.0587 (15)	0.0017 (10)	−0.0016 (10)	0.0162 (12)
C5	0.0377 (10)	0.0573 (15)	0.0632 (15)	0.0004 (10)	0.0066 (10)	0.0057 (11)
C6	0.0419 (10)	0.0420 (12)	0.0477 (12)	−0.0026 (9)	0.0087 (8)	−0.0025 (9)
C7	0.0417 (11)	0.0477 (14)	0.0526 (13)	−0.0010 (9)	0.0114 (10)	−0.0009 (10)
C8	0.0430 (12)	0.0476 (13)	0.0466 (12)	−0.0036 (9)	0.0130 (9)	−0.0022 (10)
C9	0.0701 (16)	0.0767 (18)	0.0617 (17)	0.0157 (14)	0.0148 (13)	0.0164 (14)
C10	0.0583 (15)	0.098 (2)	0.081 (2)	−0.0028 (14)	0.0332 (14)	0.0230 (16)
N1	0.0457 (9)	0.0486 (11)	0.0459 (9)	−0.0044 (8)	0.0120 (8)	0.0006 (9)
N2	0.0388 (9)	0.0593 (12)	0.0544 (12)	−0.0024 (8)	0.0135 (8)	0.0107 (9)
O1	0.0429 (8)	0.0631 (10)	0.0591 (10)	0.0033 (8)	0.0050 (7)	0.0137 (8)
O2	0.0433 (8)	0.0628 (9)	0.0653 (10)	0.0004 (7)	0.0185 (7)	0.0103 (8)
O3	0.0413 (9)	0.0889 (13)	0.0689 (11)	0.0005 (8)	0.0122 (7)	0.0093 (10)
O4	0.0499 (8)	0.0641 (11)	0.0596 (10)	0.0070 (7)	0.0151 (7)	0.0183 (8)

### Geometric parameters (Å, °)

**Table:**

C1—O1	1.361 (2)	C8—O3	1.192 (3)
C1—C6	1.399 (3)	C8—O4	1.347 (3)
C1—C2	1.404 (3)	C8—N2	1.349 (3)
C2—O2	1.367 (3)	C9—O4	1.438 (3)
C2—C3	1.382 (3)	C9—H9A	0.96
C3—C4	1.392 (3)	C9—H9B	0.96
C3—H3	0.93	C9—H9C	0.96
C4—C5	1.363 (4)	C10—O2	1.423 (3)
C4—H4	0.93	C10—H10A	0.96
C5—C6	1.405 (3)	C10—H10B	0.96
C5—H5	0.93	C10—H10C	0.96
C6—C7	1.460 (3)	N1—N2	1.374 (2)
C7—N1	1.277 (3)	N2—H2	0.86
C7—H7	0.93	O1—H1	0.82
			
O1—C1—C6	123.41 (19)	O3—C8—N2	125.9 (2)
O1—C1—C2	116.80 (17)	O4—C8—N2	109.70 (18)
C6—C1—C2	119.79 (19)	O4—C9—H9A	109.5
O2—C2—C3	125.2 (2)	O4—C9—H9B	109.5
O2—C2—C1	114.75 (19)	H9A—C9—H9B	109.5
C3—C2—C1	120.00 (19)	O4—C9—H9C	109.5
C2—C3—C4	119.8 (2)	H9A—C9—H9C	109.5
C2—C3—H3	120.1	H9B—C9—H9C	109.5
C4—C3—H3	120.1	O2—C10—H10A	109.5
C5—C4—C3	120.9 (2)	O2—C10—H10B	109.5
C5—C4—H4	119.6	H10A—C10—H10B	109.5
C3—C4—H4	119.6	O2—C10—H10C	109.5
C4—C5—C6	120.5 (2)	H10A—C10—H10C	109.5
C4—C5—H5	119.8	H10B—C10—H10C	109.5
C6—C5—H5	119.8	C7—N1—N2	118.03 (16)
C1—C6—C5	119.0 (2)	C8—N2—N1	117.36 (17)
C1—C6—C7	122.28 (19)	C8—N2—H2	121.3
C5—C6—C7	118.7 (2)	N1—N2—H2	121.3
N1—C7—C6	120.31 (18)	C1—O1—H1	109.5
N1—C7—H7	119.8	C2—O2—C10	116.9 (2)
C6—C7—H7	119.8	C8—O4—C9	114.74 (19)
O3—C8—O4	124.4 (2)		

### Hydrogen-bond geometry (Å, °)

**Table:**

*D*—H···*A*	*D*—H	H···*A*	*D*···*A*	*D*—H···*A*
O1—H1···N1	0.82	1.93	2.645 (2)	145
N2—H2···O2^i^	0.86	2.42	3.022 (2)	127
C5—H5···O3^ii^	0.93	2.49	3.320 (2)	149
C7—H7···O3^ii^	0.93	2.45	3.291 (2)	150
C3—H3···Cg1^iii^	0.93	2.85	3.606 (2)	139

Symmetry codes: (i) *x*−1/2, −*y*+1/2, *z*+1/2; (ii) *x*−1/2, −*y*+1/2, *z*−1/2; (iii) *x*, −*y*+1, *z*−1/2.
